# Effects of atorvastatin loading prior to primary percutaneous coronary intervention on endothelial function and inflammatory factors in patients with ST-segment elevation myocardial infarction

**DOI:** 10.3892/etm.2013.1432

**Published:** 2013-11-28

**Authors:** HUIJUAN YONG, XIN WANG, LIN MI, LIJUN GUO, WEI GAO, YONGZHEN ZHANG, MING CUI

**Affiliations:** 1 Department of Cardiology, Peking University Third Hospital, Beijing 100191, P.R. China; 2 Key Laboratory of Cardiovascular Molecular Biology and Regulatory Peptides, Ministry of Health and Key Laboratory of Molecular Cardiovascular Sciences, Ministry of Education, Beijing 100191, P.R. China

**Keywords:** ST-segment elevation myocardial infarction, primary percutaneous coronary intervention, atorvastatin, endothelial function, inflammatory reaction

## Abstract

Previous studies have demonstrated the beneficial effect of statin loading prior to elective and early percutaneous coronary intervention (PCI), in which the ‘pleiotropic effects’ of statins may contribute to these clinical benefits. The aim of the present study was to examine the potential effects of atorvastatin loading prior to primary PCI on coronary endothelial function and inflammatory factors in patients with acute ST-segment elevation myocardial infarction (STEMI). A total of 60 patients with STEMI were randomized into three groups: Loading dose (80 mg atorvastatin prior to PCI; n=20), regular dose (20 mg atorvastatin prior to PCI; n=20) and control (without atorvastatin prior to PCI; n=20). The plasma samples were collected prior to, and immediately, 6 and 24 h after PCI in all the patients. The plasma concentrations of endothelial nitric oxide synthase (eNOS), nitric oxide (NO), interleukin-6 (IL-6), tumor necrosis factor-α (TNF-α) and intercellular adhesion molecule-1 (ICAM-1) were examined using ELISA. The plasma eNOS levels immediately and 24 h after PCI were significantly higher in the regular dose group compared with the other groups. However, there were no significant differences in the plasma eNOS concentration prior to and 6 h after PCI, or in the plasma NO concentration at any of the time-points among the three groups. The plasma IL-6 levels prior to PCI were significantly lower in the loading dose group compared with the other groups; however, there were no significant differences in the plasma concentration of IL-6 following PCI or in the concentrations of TNF-α and ICAM-1 at any of the time-points among the three groups. In conclusion, atorvastatin loading in patients with STEMI undergoing primary PCI may not have protective effects on endothelial function and the inflammatory reaction.

## Introduction

Previous large-scale studies have demonstrated the beneficial effects of statin loading prior to elective and early percutaneous coronary intervention (PCI) for the prevention of major adverse cardiac events (MACEs), including angina pectoris, mortality, nonfatal myocardial infarction (MI) and target vessel revascularization, as well as stable angina pectoris (SAP), unstable angina pectoris (UAP) and non-ST-segment elevation myocardial infarction (NSTEMI) (1–10). The ‘pleiotropic effects’ of statins include the modulation of endothelial function, inhibition of inflammation and attenuation of thrombosis, all of which can provide clinical benefits for elective and early PCI via reductions in the postprocedural incidence of MI and MACEs.

However, little is known with regard to the effect of statin loading prior to primary PCI in patients with acute ST-segment elevation myocardial infarction (STEMI). Previous observational studies on patients with STEMI have suggested that chronic previous statin use may improve coronary blood flow, and is associated with reduced short-term (30-day) mortality (11–14). However, the beneficial effects of chronic statin pretreatment have limitations in their applicability due to the unexpected nature of the onset of acute STEMI, whereas the acute effects of high-dose statins may be more clinically relevant in the emergent setting in STEMI. In a retrospective cohort study, statin therapy at the time of primary PCI for STEMI and cardiogenic shock was associated with a significant mortality advantage at early follow-up (15). The STATIN STEMI trial (16) was a randomized, prospective study, which demonstrated that high-dose atorvastatin pretreatment prior to primary PCI did not lead to a significant reduction in MACEs compared with low-dose atorvastatin. However, the study showed improved immediate coronary flow following primary PCI. Another randomized controlled study demonstrated that pretreatment with high-dose atorvastatin, followed by further treatment for five days, did not reduce infarct size, measured by single-photon emission computed tomography, in patients undergoing primary PCI (17). By contrast, a recent study showed that preprocedural high-dose atorvastatin prevented contrast-induced nephropathy (CIN) and protected renal function in patients with acute STEMI undergoing primary PCI (18). However, to date, the efficacy of atorvastatin loading in patients with STEMI undergoing primary PCI has not been demonstrated. In addition, it has not yet been elucidated whether the ‘pleiotropic effects’ of statins can explain the possible mechanism(s) behind the action of statins.

Therefore, the aim of this prospective randomized trial was to examine the efficacy of high-dose atorvastatin immediately prior to primary PCI on coronary endothelial function and inflammation in patients with STEMI.

## Patients and methods

### Patient selection

This study was a randomized, prospective clinical trial and was approved by the Ethics Review Boards of Peking University Third Hospital (Beijing, China). All patients provided consent for a sample of their blood to be used for scientific purposes.

The inclusion criteria were as follows: STEMI diagnosed according to the 2004 American College of Cardiology/American Heart Association guidelines; patients receiving primary PCI within 12 h from symptom onset; and Thrombolysis In Myocardial Infarction (TIMI) flow grade ≥2 at the end of the procedure. The exclusion criteria comprised: Patients aged >80 or <18 years; cardiogenic shock or severe heart failure on admission; patients receiving electric defibrillation, a temporary pacemaker or intra-aortic balloon pump (IABP) during the PCI; TIMI flow grade <2 at the end of the procedure; previous history of MI or PCI; previous (within three months) or current treatment with statins; known allergy to statins; and chronic inflammatory, significant kidney or hepatic diseases, tumor, myositis or myopathy.

A total of 80 consecutive patients with STEMI admitted to the Department of Cardiology, Peking University Third Hospital, between October 2010 and June 2011, were included in the study. Of the 80 patients, 6 patients were excluded due to previous (within three months) or current treatment with statins, 5 patients received a temporary pacemaker and/or IABP during PCI, 4 patients had a previous history of MI or PCI, 2 patients underwent a percutaneous transluminal coronary angioplasty instead of PCI, 3 patients received electric defibrillation and 1 patient was >80 years. The eligible patients (n=60) were randomized into three groups: Loading dose (80 mg atorvastatin prior to PCI; n=20), regular dose (20 mg atorvastatin prior to PCI; n=20) and control (without atorvastatin prior to PCI; n=20).

### Treatment and procedures

All patients were pretreated with a loading dose of aspirin (300 mg) and clopidogrel (300–600 mg) at the emergency department prior to intervention. The patients were administered with weight-adjusted intravenous heparin at 100 U/kg in the absence of glycoprotein IIb/IIIa inhibitor therapy and 70 U/kg with glycoprotein IIb/IIIa inhibitor. Glycoprotein IIb/IIIa inhibitors and thrombus aspiration were used during the procedure at the discretion of the surgeon. The PCI procedure was performed according to standard technique (19). Following PCI, the patients were administered with standard therapy, including aspirin (100 mg/day) indefinitely, clopidogrel (75 mg/day) for ≥1 year, atorvastatin (20 mg/day), β-blockers and angiotensin-converting enzyme (ACE) inhibitors if there were no contraindications, irrespective of the initial randomization assignment.

### Laboratory assays

Venous blood samples were collected from all patients prior to, and immediately, 6 and 24 h after PCI. All samples were collected into vacuum blood collection tubes with EDTA and were immediately placed in refrigerators at 4°C. Within 30 min after collection, the samples were centrifuged at 1,000 × g for 10 min at 4°C, divided into aliquots and stored at −80°C. Repeated freeze-thaw cycles were avoided.

Plasma concentrations of endothelial nitric oxide synthase (eNOS), nitric oxide (NO), interleukin-6 (IL-6), tumor necrosis factor-α (TNF-α) and intercellular adhesion molecule-1 (ICAM-1) were measured using ELISA, in accordance with the manufacturer’s instructions (ELISA kit; R&D Systems, Minneapolis, MN, USA). The upper and lower detection limits, were 100 and 1.56 U/ml for eNOS and NO, 300 and 4.7 pg/ml for IL-6, 1,000 and 15.6 pg/ml for TNF-α, and 1.0×10^4^ and 156 pg/ml for ICAM-1, respectively. The sensitivities were <0.3 U/ml for eNOS and NO, <0.3 pg/ml for IL-6, <1.5 pg/ml for TNF-α and <15 pg/ml for ICAM-1. The assays were performed by an investigator who was blinded to the source of the samples.

In all patients, the blood samples were collected whenever it was possible prior to and 2, 6, 12, 24, 48, 72 h post-PCI to measure the serum creatine kinase-myocardial band (CK-MB) isoenzyme and troponin T elevation; other measurements were performed in cases of post-procedural symptoms suggestive of myocardial ischemia. Normal limits of CK-MB and troponin T were defined as ≤24 U/l and ≤0.1 ng/ml, respectively. Liver function and the levels of high-sensitivity C-reactive protein (hs-CRP) and amino terminal-pro brain natriuretic peptide (NT-proBNP) were evaluated 12–24 h after primary PCI.

### Coronarography, electrocardiographic analysis and echocardiography

The TIMI flow grade and Gensini Score were analyzed by two experienced scientists (20,21). The electrocardiograms (ECGs) were read prior to and 60 min post-primary PCI by one physician. ST-segment resolution was calculated as the maximum ST-segment elevation on the initial ECG minus the ST-segment elevation of the same lead on the ECG at 60 min post-PCI, divided by the maximum ST-segment elevation on the initial ECG, expressed as a percentage (22). The echocardiography evaluation was performed by specialist physicians.

### Clinical follow-up

A six-month clinical follow-up was completed for all patients to evaluate the incidence of MACEs and the safety of atorvastatin loading.

### Statistical analysis

All analyses were performed with SPSS 19.0 statistical software (SPSS Inc, Chicago, IL, USA). The Kolmogorov-Smirnov test was used to assess the normal distribution of continuous variables. Normally distributed homogeneous data were compared using one-way analysis of variance for >2 groups, otherwise a rank sum test was performed. Proportions were compared using the χ^2^ test. P<0.05 (two-tailed) was considered to indicate a statistically significant difference. The data are expressed numerically (as a percentage), as the mean ± standard deviation or as the median (minimum; maximum), as appropriate.

## Results

### General characteristics

The clinical characteristics are summarized in Table I. There were no significant differences in any of the clinical parameters with medications, such as aspirin, clopidogrel, β-blockers or ACE inhibitors.

The coronary-angiography characteristics are summarized in Table II. There were no significant differences in the studied coronarography parameters. There were also no significant differences in the laboratory results, including glucose, glycosylated hemoglobin, triglyceride, total cholesterol, low-density lipoprotein cholesterol, creatinine and complete blood count, with the exception of the high-density lipoprotein cholesterol levels (data not shown).

### Plasma eNOS and NO

**Figure 1:**
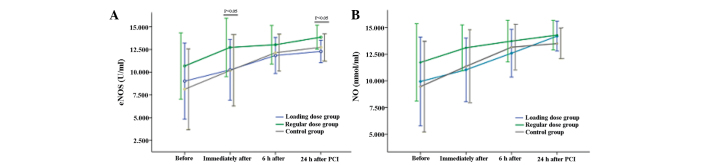
Levels of plasma endothelial function factors in various groups. Plasma (A) eNOS and (B) NO levels. The plasma eNOS levels immediately following and 24 h post-PCI were significantly higher in the regular dose group than the other groups. There were no significant differences in the plasma eNOS levels prior to and 6 h post-PCI, or in the plasma NO levels at any of the time-points among the three groups. eNOS, endothelial nitric oxide synthase; NO, nitric oxide; PCI, percutaneous coronary intervention.

Plasma eNOS and NO levels from the three groups at four time-points are shown in Fig. 1. The plasma eNOS levels immediately (12.73±3.22 vs. 10.26±3.35 vs. 10.19±3.93 for regular dose group, loading dose group and control group, respectively; P=0.026) and 24 h (13.86±1.33 vs. 12.28±1.24 vs. 12.74±1.46 for regular dose group, loading dose group and control group, respectively; P=0.002) post-PCI were significantly higher in the regular dose group compared with the other two groups. However, there were no significant differences in the plasma eNOS concentrations prior to and 6 h post-PCI, or in the plasma NO concentration at any of the time-points among the three groups (P>0.05).

### Plasma IL-6, TNF-α and ICAM-1

**Figure 2:**
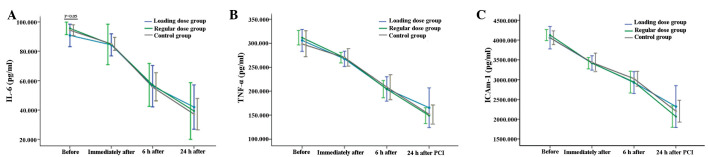
Levels of plasma inflammatory factors in various groups. Plasma (A) IL-6, (B) TNF-α and (C) ICAM-1 levels. The plasma concentration of IL-6 prior to PCI was significantly lower in the loading dose group than the other groups. There were no significant differences in the plasma levels of IL-6 post-PCI or in the plasma levels of TNF-α and ICAM-1 at any of the time-points among the three groups. IL-6, interleukin-6;TNF-α, tumor necrosis factor-α; ICAM-1, intercellular adhesion molecule-1; PCI, percutaneous coronary intervention.

Plasma IL-6, TNF-α and ICAM-1 levels of the three groups at four time-points are shown in Fig. 2. The plasma IL-6 levels prior to PCI were significantly lower in the loading dose group compared with the other two groups (90.77±7.65 vs. 95.59±4.27 vs. 94.32±3.69 for loading dose group, regular dose group and control group, respectively; P=0.023). However, there were no significant differences in the plasma IL-6 concentration following PCI or in the plasma TNF-α and ICAM-1 concentrations at any of the time-points among the three groups (P>0.05).

### Clinical efficacy index

The clinical efficacy indices are shown in Table III. Peak CK-MB, hs-CRP, NT-proBNP, electrocardiograph ST-segment resolution at 60 min and echocardiography did not show any significant differences among the three groups (P>0.05). MACEs occurred in 2 patients (10.0%) in the loading dose group, 2 patients (10.0%) in the regular dose group and 3 patients (15.0%) in the control group, respectively (P>0.05).

### Safety of atorvastatin loading

Patient liver function prior to being discharged did not show any significant differences among the three groups (P>0.05). None of the patients suffered from myalgia during the study.

## Discussion

Endothelium-derived NO can mediate vascular smooth muscle relaxation (23), inhibit platelet activation (24) and the proliferation of vascular smooth muscle cells (25), and protect against leukocyte-endothelium interactions (26,27); therefore, it exhibits anti-atherosclerotic effects. Statin treatment improves endothelium-dependent coronary vasomotion within 24 h in the absence of significant cholesterol reduction (28–31). Furthermore, statins upregulate eNOS expression (32) and increase the production of endothelium-derived NO. To the best of our knowledge, the present study is the first to demonstrate that atorvastatin loading in patients with STEMI undergoing primary PCI may not exert protective effects on endothelial function. This may be attributed to heavily damaged endothelial function in patients with STEMI and the damage may be too severe for a single dose of atorvastatin in a short time (~1.2 h) to elicit any improvement. However, vascular endothelial function was only assessed via the measurements of plasma eNOS and NO levels, rather than by the direct observation of the relaxation and contraction of the coronary artery.

Previous studies have demonstrated that a number of inflammatory factors are involved in the course of coronary heart disease, such as IL, TNF-α and ICAM-1. An animal experiment revealed that arterial injury in human CRP-transgenic mice resulted in an expedited and higher rate of thrombotic occlusion (33). Another study showed that there were significant differences in the TNF-α IL-6 and CRP levels of patients who were troponin T-positive versus patients who were troponin T-negative following selective PCI (34). The extent of inflammation may affect the prognosis of patients post-PCI. Patients with a higher degree of inflammatory cell activation are more likely to suffer from coronary artery restenosis. However, the inflammatory reaction triggered by PCI is not limited to the position of stents and may spread to surrounding tissue, including the myocardial layer. Furthermore, a higher level of inflammatory reaction is also capable of exacerbating the lesions of the arteries (35). The Atorvastatin for Reduction of Myocardial Damage during Angioplasty (ARMYDA) (6), ARMYDA-acute coronary syndromes (8), ARMYDA-RECAPTURE (10) and Novel Approaches for Preventing or Limiting Events II (9) studies demonstrated that pretreatment with atorvastatin significantly reduced procedural CRP levels of patients with SAP, UAP and NSTEMI in elective coronary intervention. The ARMYDA during Angioplasty-Cell Adhesion Molecules (ARMYDA-CAMS) study (36) revealed that, in patients undergoing PCI, a reduction in procedural myocardial injury following a seven-day pretreatment regimen with atorvastatin was paralleled by a concomitant attenuation of post-procedural increases in ICAM-1 and E-selectin levels. Thus, it was suggested that a reduction in the endothelial inflammatory response may explain the protective effect of statins (36). Another study indicated that the administration of a single dose of cerivastatin to patients with UAP or NSTEMI at the time of admission was capable of decreasing the serum level of CRP and IL-6 24 h later (37). In contrast with these previous studies, the present study is the first to demonstrate that atorvastatin loading in patients with STEMI undergoing primary PCI may not decrease the inflammatory response. This may be associated with the more severe inflammatory reaction in patients with STEMI, which was thus not capable of being alleviated by a single dose of atorvastatin in a short time (~1.2 h). In addition, only three inflammatory factors (IL-6, TNF-α and ICAM-1) were observed in the study. There are numerous other inflammatory factors involved in the inflammatory response in coronary heart disease that were not studied in the present investigation, such as IL-1 and E-selectin. In the STATIN STEMI trial, 171 patients were randomized to two groups receiving pretreatment with 80 mg atorvastatin (n=86) or 10 mg atorvastatin (n=85) prior to PCI. There was no difference in the CRP levels at 24 h post-PCI between the two groups, which was consistent with the results of the present study.

The efficacy of atorvastatin loading in patients with STEMI undergoing primary PCI has not been confirmed. If it does exhibit clinical benefit, the mechanism underlying the effects, and whether the ‘pleiotropic effects’ of statins are capable of explaining the possible mechanism(s) have yet to be elucidated. The present study examined the potential effects of atorvastatin loading prior to primary PCI on coronary endothelial function and inflammatory factors in patients with STEMI. According to present protocol, patients are administered with 300 mg asprin (100 mg/per pill) and 300–600 mg clopidogrel (75 mg/per pill) pretreatment prior to primary PCI. As a result, patients are required to take 7–11 pills. If high-dose atorvastatin pretreatment prior to PCI does not lead to a significant reduction in MACEs, the atorvastatin pretreatment is unnecessary. This may result in a reduction in doses, cost and side effects, for example gastrointestinal discomfort.

To the best of our knowledge, the present study is the first randomized trial to examine the potential effects of atorvastatin loading prior to primary PCI on coronary endothelial function and inflammatory factors in patients with STEMI. However, there were certain limitations to the study which require further investigation. The study sample size was not large enough to evaluate the efficacy of high-dose atorvastatin loading (80 mg) prior to primary PCI in STEMI. Furthermore, the effects of 80-mg atorvastatin pretreatment on other ‘pleiotropic effects’, including antithrombosis, antiarrhythmia and the prevention of CIN, and the efficacy of pretreatment with other statins prior to primary PCI, were not investigated.

In conclusion, atorvastatin loading in patients with STEMI undergoing primary PCI may not have protective effects on endothelial function, inflammation, cardiac perfusion, heart function or MACEs; however, it did not result in damage to the liver or muscles.

## Figures and Tables

**Table I:** Baseline clinical characteristics.

Parameter	Loading dose group (n=20)	Regular dose group (n=20)	Control group (n=20)	P-value
Age, years	54.5±12.7	61.5±11.7	55.6±7.8	0.119
Male, %	90.0	75.0	80.0	0.437
Abdominal girth, cm	95.8±13.7	92.1±9.1	93.5±8.0	0.550
Diabetes mellitus, %	20.0	10.0	25.0	0.437
Hyperlipidemia, %	40.0	25.0	15.0	0.198
Hypertension, %	60.0	60.0	55.0	0.934
Stroke, %	15.0	25.0	10.0	0.436
Current smoker, %	75.0	70.0	85.0	0.508
CHD family history, %	20.0	10.0	20.0	0.597
Anterior MI, %	55.0	45.0	60.0	0.626
Killip classification >I, %	10.0	5.0	5.0	1.000
Time from symptom onset to PCI, h	4.2 (2.3;12.0)	4.0 (1.0;12.5)	3.9 (1.5;31.5)	0.998

Data are expressed numerically (as a percentage), as the mean ± standard deviation or as the median (minimum; maximum), as appropriate. CHD, coronary heart disease; MI, myocardial infarction; PCI, percutaneous coronary intervention.

**Table II:** Coronarography characteristics.

Parameter	Loading dose group (n=20)	Regular dose group (n=20)	Control group (n=20)	P-value
Counts				
Single vessel, %	35.0	30.0	20.0	0.396
Double vessel, %	40.0	20.0	40.0	0.396
Triple vessel, %	25.0	50.0	40.0	0.396
Culprit vessel				
LAD, %	60.0	45.0	60.0	0.471
LCX, %	10.0	10.0	20.0	0.471
RCA, %	30.0	45.0	20.0	0.471
Gensini score	47.3 (24.0;106.0)	58.3 (20.0;98.5)	53.0 (14.0;92.0)	0.720
TIMI prior to PCI, %	70.0	75.0	80.0	0.766
Stent=1, %	85.0	90.0	95.0	0.561
Collateral formation, %	10.0	20.0	30.0	0.273
Periprocedural arrhythmia, %	45.0	25.0	20.0	0.189
Glycoprotein IIb/IIIa inhibitor therapy, %	30.0	45.0	60.0	0.162

Data are expressed numerically (as a percentage), as the mean ± standard deviation or as the median (minimum; maximum), as appropriate. LAD, left anterior descending artery; LCX, left circumflex artery; RCA, right coronary artery; TIMI, thrombolysis in myocardial infarction; PCI, percutaneous coronary intervention.

**Table III:** Clinical efficacy index in various groups.

Parameter	Loading dose group (n=20)	Regular dose group (n=20)	Control group (n=20)	P-value
Peak CK, U/l	1877 (632;8927)	1600 (820;6229)	1607 (275;5221)	0.502
Peak CK-MB, U/l	240 (91;720)	209 (113;900)	246 (33;741)	0.558
hs-CRP, mg/l	5.98 (0.68;29.74)	7.21 (1.17;50.36)	6.22 (1.10;117.44)	0.651
NT-proBNP, pg/ml	1005 (24;7699)	1047 (83;3705)	1049 (102;13839)	0.994
ST-segment resolution, %	63±37	65±31	52±35	0.464
LVESD, mm	36.6±7.0	35.9±7.4	35.2±3.5	0.729
LVEDD, mm	49.8±5.4	47.7±9.9	49.9±3.2	0.507
LVEF, %	51±7	51±8	53±7	0.501
Left atrial area, mm^2^	19.2±2.9	20.1±3.3	20.1±4.1	0.618
Left atrial diameter, mm	37.1±3.2	35.2±4.1	36.5±4.4	0.326
LAP, mmHg	12±3	12±4	14±6	0.399
MACEs, %	10	10	15	0.855
Angina pectoris, %	10	10	15	
Nonfatal MI, %	0	0	0	
Mortality, %	0	0	0	
Target vessel revascularization, %	0	0	0	

Data are expressed numerically (as a percentage), as the mean ± standard deviation or as the median (minimum; maximum), as appropriate. CK, creatine kinase; CK-MB, creatine kinase-myocardial band; hs-CRP, high-sensitivity C-reactive protein; NT-proBNP, amino terminal-pro brain natriuretic peptide; LVESD, left ventricular end systolic diameter; LVEDD, left ventricular end diasystolic diameter; LVEF, left ventricular ejection fraction; LAP, left atrial pressure; MACEs, major adverse cardiac events; MI, myocardial infarction.
